# COVID-19 Vaccine-Induced Expansion of Pituitary Adenoma: A Case Report

**DOI:** 10.7759/cureus.50685

**Published:** 2023-12-17

**Authors:** Worapot Srimanan, Somboon Panyakorn

**Affiliations:** 1 Ophthalmology, Phramongkutklao Hospital, Bangkok, THA

**Keywords:** visual field, visual loss, pituitary adenoma, vaccine, covid-19

## Abstract

A pituitary adenoma is an insidious and slow-growing neoplasm of the pituitary gland. No definitive aggravating factors have currently been reported for pituitary adenoma enlargement. Our case demonstrates that the coronavirus disease 2019 (COVID-19) vaccine may be one of the risk factors aggravating tumor expansion.

A 60-year-old woman experienced visual loss in her left eye for three days. Eight days before presentation or five days before visual loss, she received the fourth dose of the COVID-19 vaccine. The visual field showed bitemporal superior quadrantanopic scotoma, prominent on the left side. Neuroimaging revealed pituitary macroadenoma with a compressive effect on the optic chiasm. After diagnosis, endocrine work-up and tumor removal were successfully performed. Her vision and perimetry significantly improved.

The COVID-19 vaccine is a candidate factor that might exacerbate pituitary gland enlargement. Additional data are essential to validate and establish the connection between the vaccine and this potential effect.

## Introduction

A pituitary adenoma is an anterior pituitary tumor, usually benign and slow-growing [1]. It could be a functioning tumor if it secretes one or more hormones from the anterior pituitary. In contrast, non-functioning tumors could compress surrounding organs, leading to effects such as headache, visual loss, visual field defect, or hormonal deficiency. Epidemiological studies have revealed an incidence of pituitary adenoma ranging from 3.9 to 7.4 cases per 100,000 per year and a prevalence of 76-116 cases per 100,000 in the general population [2].

Both the coronavirus disease 2019 (COVID-19) virus and its vaccine impact the endocrine system. A COVID-19 infection is associated with various endocrine issues such as disruptions in glucose regulation, thyroid dysfunction, adrenal insufficiency, and changes in the hypothalamic-pituitary axis [3]. Conversely, the vaccine has rarely been linked to transient endocrine-related complications like thyroiditis, pancreatitis, and occasional pituitary gland enlargement [4]. These vaccine-induced effects, notably on the pituitary gland, include reports of pituitary apoplexy [5-9] or, less frequently, pituitary hypophysitis [10,11] in the literature.

Currently, no evidence supports the risk factors for expanding the size of pituitary tumors [1]. Herein, we report an interesting case that supports the COVID-19 vaccine as one of the possible risk factors aggravating this condition. The case involves a 60-year-old female with acute visual loss from expanding pituitary adenoma after COVID-19 vaccination.

## Case presentation

A 60-year-old woman experienced sudden vision loss in her left eye three days after receiving her fourth dose of the Pfizer-BioNTech COVID-19 vaccine. After vaccination, she had minimal constitutional symptoms, including myalgia, shoulder pain, and low-grade fever for only two days. She had allergic asthma and seasonal allergic rhinitis in her past medical history. She visited an outpatient department every six months for primary angle closure suspect with peripheral iridotomy in both eyes, best corrected visual acuity (BCVA) 20/32 in her right eye, and 20/30 in her left eye two months before vaccination.

**Figure 1:**
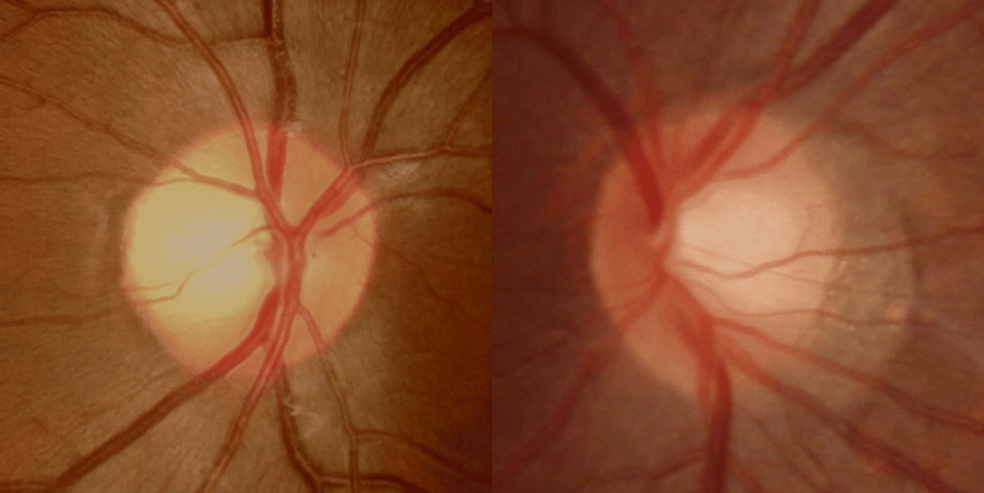
Optic disc photography of the patient, right eye (left figure) and left eye (right figure) The picture was taken on the first visit to the outpatient department.

She denied pain in eye movement, double vision, or any accompanying symptoms at the outpatient department. Her BCVA was 20/25 in her right eye and 20/125 in her left eye, respectively. The Ishihara test revealed normal color vision in her right eye with a color deficit in her left eye. The ocular examination was regular, except for a relative afferent pupillary defect in her left eye (grade 2). The optic discs were sharp and pink, with mild temporal pallor, and a cup-to-disc ratio of 0.6 in both eyes, as shown in Figure 1.

**Figure 2:**
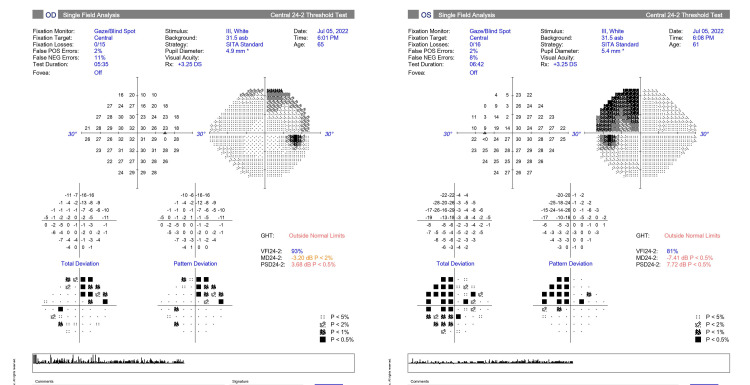
Visual field 24–2 automated perimetry demonstrated bitemporal superior quadrantanopic scotoma, more prominent in the left eye (right figure) The visual field was taken in the outpatient department on the date of the patient’s first visit.

The visual field test in Figure 2 revealed bitemporal superior quadrantanopia, more prominent in the left eye.

The etiologies of acute optic neuropathy could be inflammation, infection, or ischemic-related. The laboratory workup included magnetic resonance imaging of the brain and orbit.

The following laboratory test results indicated a normal complete blood count: white blood cells, 8600/µL (4,500-10,500); polymorphonuclear leukocytes, 72.6% (25-75); lymphocytes, 22.5% (20-40); and platelets, 346,000/µL (162,000-402,000). These results also revealed an unremarkable erythrocyte sedimentation rate of 4 mm/hr (0-20) and a C-reactive protein level of 3.5 mg/L (0-5).

**Figure 3:**
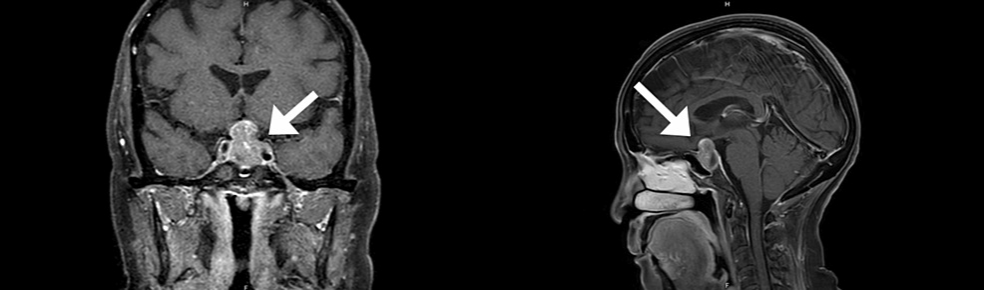
Coronal T1 (left figure) and sagittal T1 view (right figure) of magnetic resonance imaging confirming macroadenoma, compressing the optic chiasm and extending to suprasellar cisterns and the left cavernous sinus (arrow).

Magnetic resonance imaging of the brain and orbit, shown in Figure 3, revealed a well-defined homogeneous enhancement of the T2 hypo/hyperintense signal of an intrasellar mass with suprasellar extension, 1.8x1.3x2.6 cm in size, abutting the bilateral supraclinoid part of the internal carotid artery. The normal pituitary gland and posterior pituitary bright spot were not demonstrated. The mass caused a pressure effect on the optic chasm. No recent intratumoral hemorrhage was observed. Such findings suggested pituitary macroadenoma.

Endocrinologists performed a workup for hormonal function. The high prolactin level was confirmed to have a stalk effect after a 1:1 dilutional test. A non-functioning pituitary tumor was diagnosed in this case (Table 1).

**Table 1:** A pituitary hormone workup Isolated hyperprolactinemia was confirmed to be a stalk effect after a dilutional test.

Investigations	Results	Normal ranges
Follicle-stimulating hormone (IU/L)	13.3	0.11-198
Luteinizing hormone (IU/L)	4.25	0.11-198
Random cortisol (mmol/L)	9.34	6.02-18.4
Morning cortisol (mmol/L)	6.95	6.02-18.4
Prolactin (mIU/L)	91.57	3.4-24.1
Thyroid-stimulating hormone (mIU/L)	1.34	0.27-4.2
Free thyroxine (pmol/L)	0.93	0.93-1.71
Insulin-like growth factor 1 (ng/mL)	136.3	40-222

The neurosurgeon performed an endoscopic endonasal procedure for tumor removal. The surgical pathology report showed low-grade neuroendocrine neoplasm, favoring a pituitary adenoma. There was no area of hemorrhage or necrosis. There was no intraoperative complication.

**Figure 4:**
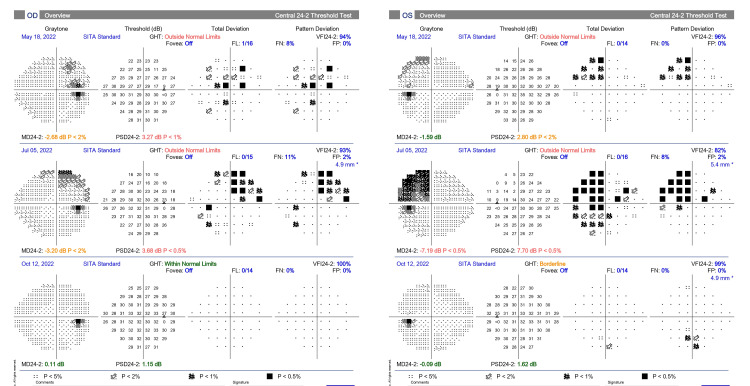
Serial 3 visual field automated perimetries: (above) before vaccination, (middle) after vaccination, and (bottom) after surgery, resolution of the visual field defect

After the operation, the patient’s vision gradually improved. One month after the procedure, her BCVA was 20/25 in her right eye and 20/40 in her left eye. Her visual field improved considerably, as shown in Figure 4. The pupillary reaction and color vision recovered. At three months, the patient’s BCVA was 20/25 in her right eye and 20/25 in her left eye. Color vision finally became normal. Six months after the surgery, her clinical condition was stable, with no residual tumor detected in the MRI.

## Discussion

The emergence of subacute visual impairment, color perception deficiencies, abnormal pupillary responses, and a pronounced visual field impairment, in comparison to the patient's status one month post-vaccination, suggests a plausible association between the COVID-19 vaccine and the development of pituitary gland enlargement. Neuroimaging confirmed the presence of an enlarged pituitary gland exerting pressure on the optic chiasm without any signs of hemorrhage, ultimately leading to the diagnosis of pituitary macroadenoma in this case. Our case presents a distinctive manifestation of pituitary gland enlargement, resulting in vision loss.

Pituitary adenomas, typically insidious and asymptomatic, are often incidentally identified during neuroimaging examinations for various medical indications [1,2]. These sporadic tumors pose a challenge in pinpointing specific factors contributing to their growth [1,2]. While instances of rapid tumor enlargement leading to compressive symptoms are infrequent, their subtle nature often results in delayed detection and clinical manifestation [1,2]. Detecting these tumors poses a challenge in routine clinical practice due to their inconspicuous nature, frequently requiring incidental discovery during unrelated medical evaluations.

The impact of the COVID-19 pandemic extends beyond respiratory complications, affecting various vital organs [3]. Although successful preventive vaccines have been developed, concerns persist regarding potential adverse effects [4]. Reports have highlighted diverse complications following COVID-19 vaccination, with a particular focus on the endocrine system [4]. Among these complications, disturbances in endocrine organs, notably involving the thyroid, pancreas, adrenal glands, and pituitary gland, have garnered attention [4]. Pituitary apoplexy, characterized by severe symptoms, such as intense headaches, diplopia, and other endocrinological abnormalities, has been observed in a significant proportion of patients experiencing endocrine disturbances post-vaccination [5-9]. Some documented cases in the literature have highlighted pre-existing pituitary adenomas preceding the onset of pituitary apoplexy events [5,7]. Additionally, a relatively rare vaccination-linked side effect is pituitary hypophysitis, marked by secondary adrenal insufficiency leading to hyponatremia, central hypothyroidism, and central hypogonadism [10-12].

The precise pathophysiological mechanisms underlying these phenomena remain incompletely understood. However, a plausible explanation is that the pituitary gland may serve as a target for severe acute respiratory syndrome coronavirus 2 (SARS-CoV-2) due to angiotensin-converting enzyme-2 (ACE-2) receptors on the surface of pituitary cells [13]. Notably, various studies conducted in both humans and animals have revealed significant ACE2 mRNA expression in the hypothalamus and pituitary cells, further supporting this hypothesis [14]. It is postulated that COVID-19 vaccination may trigger inflammatory or immune responses that lead to endothelial dysfunction, resulting in hyperpermeability. Given the extensive and delicate vascular network within the pituitary gland, it becomes particularly susceptible to this type of reaction [15]. Subsequent edema increases pituitary size [16]. Moreover, an immune response may also be initiated by the mRNA component of the vaccine itself, potentially causing inflammation and impairing vascular function [17,18]. These combined processes culminate in a compressive effect on adjacent organs.

Upon retrospective review, identifying the mild temporal pallor of the optic disc might serve as a potential indicator for a scotoma in the previous visual field. This underscores the importance of clinical suspicion, even in cases where the visual field appears stable over time. It highlights the subtle clues that may be overlooked, necessitating a heightened awareness among clinicians. Such findings suggest the need for neuroimaging evaluation to further investigate and delineate any underlying pathology, particularly in cases where clinical suspicion arises despite seemingly stable visual fields.

The COVID-19 vaccine holds significant promise for preventing severe COVID-19 outcomes, particularly in immunocompromised individuals. Reports of endocrine complications, particularly impacting the pancreas and thyroid, have emerged. Notably, the pituitary gland, a relatively rare target, has also been implicated. Additional research is required to elucidate the precise pathophysiological relationship between the COVID-19 vaccine and pituitary gland disorder [19]. Individuals receiving the vaccine should be aware of potential side effects and promptly seek medical attention in case of serious adverse events.

## Conclusions

Pituitary gland tumors are slowly growing, primarily asymptomatic, with incidental findings. Early detection, reduced aggravating factors, and specific treatment are essential. The COVID-19 vaccine represents a novel potential contributor to the enlargement of the pituitary gland. Individuals with preexisting pituitary adenomas should be particularly vigilant regarding the possible side effects associated with this vaccine.
